# Supplementary material for: Precision Engineering of an Anti-HLA-A2 Chimeric Antigen Receptor in Regulatory T Cells for Transplant Immune Tolerance
Source: Front Immunol. 2021 Sep 20;12:686439. doi: 10.3389/fimmu.2021.686439 (PMC8488356; doi:10.3389/fimmu.2021.686439)
Supplement: Supplementary file 1 [file DataSheet_1.docx]

Supplementary Material

## Supplementary Figures

**Supplementary Figure 1: Phage-displayed SN607D8 scFv binding to HLA-A2- and HLA-A28-expressing cell lines.** The scFv from the anti-HLA-A2 hybridoma SN607D8 was cloned into a phage vector and expressed on the surface of the phage. The HLA-A2^+^HLA-A28^–^ THP-1 cells or the HLA-A2^–^HLA-A28^+^ RPMI 8226 cells were incubated with 10^7^ phages displaying the SN607D8 scFv. The cells were then stained with a biotinylated anti-phage antibody, followed by fluorochrome-conjugated streptavidin. Cell-bound phage was then analyzed using flow cytometry. Cells not incubated with phage or incubated with control phage were similarly stained and included as controls.

**
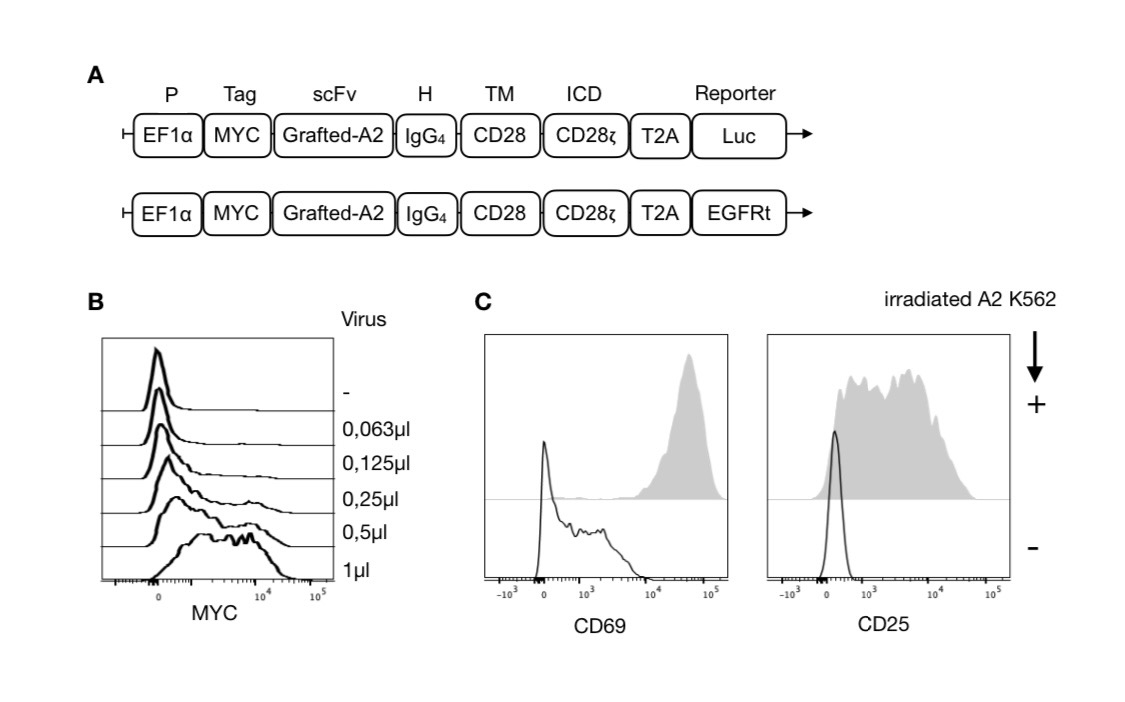
Supplementary Figure 2: Construction of a novel A2-CAR and evaluation in Jurkat T cells.** **(A)** Map of the grafted anti-HLA-A2 (A2) CAR gene fused to a luciferase (luc) or an EGFRt reporter gene. **(B)** A2-CAR lentivirus was titered on 1 x 10^4^ Jurkat T cells. CAR surface expression was assessed by MYC-tag staining 72 hours post-transduction. **(C)** 2.5 x 10^4^ A2-CAR transduced Jurkat T cells were co-cultured with irradiated (4000 rad) GFP^+^ K562 cells expressing HLA-A2 at a 1:1 ratio for 24 hours. CD69 and CD25 expression were analyzed using flow cytometry. As a control, A2-CAR Jurkat T cells were incubated alone. *Abbreviations: P, promoter; H, hinge; TM, transmembrane; ICD, intracellular domain; Luc, luciferase; A2, HLA-A2.*


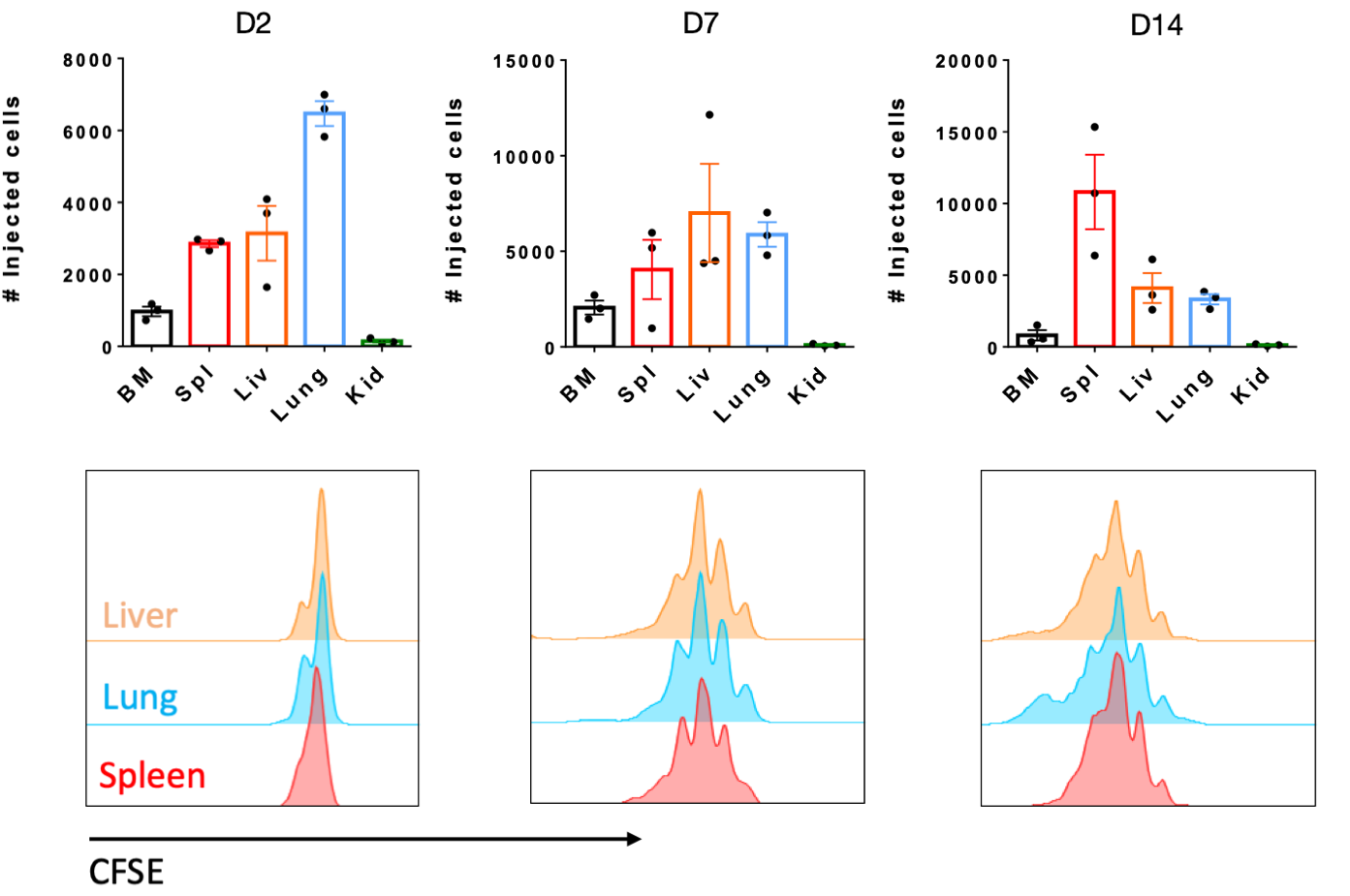


**Supplementary Figure 3: Human CD4^+^ Tconv cell trafficking and accumulation in NSG mice***.* 5 x 10^6^ CFSE-labeled human CD4^+^ Tconv cells were injected i.v. into NSG mice. On Day 2, 7, and 14 after cell transfer, their presence (top row) and proliferation (bottom histograms) in the bone marrow (BM), spleen (Spl), liver (Liv), lungs, and kidneys (Kid) of the recipient mice were analyzed using flow cytometry.


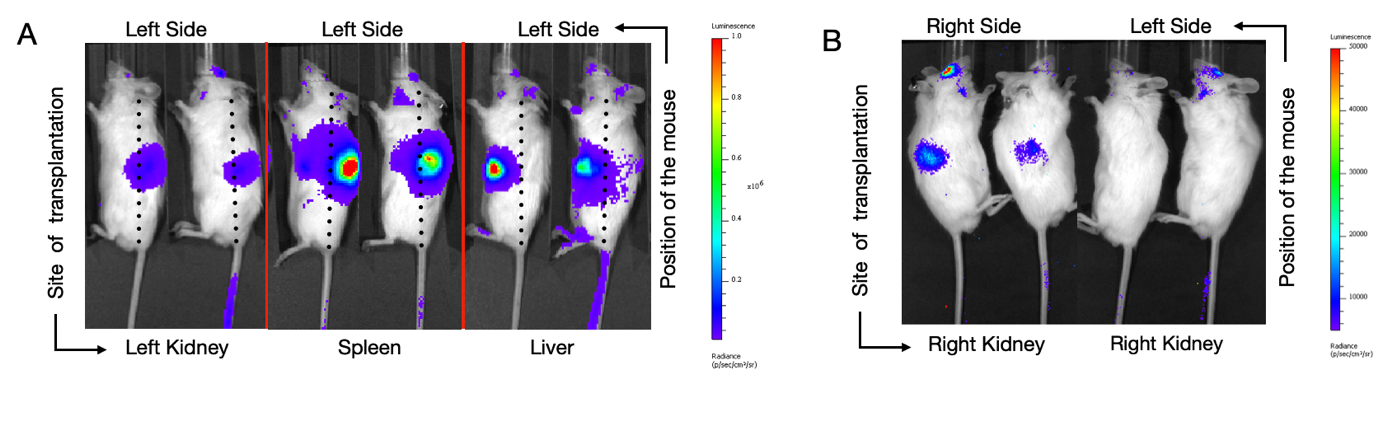


**Supplementary Figure 4: Human A2-CAR T cell trafficking to and accumulation at HLA-A2-expressing islet transplant sites in NSG mice.** A2-CAR T cells engineered using cells from HLA-A2-negative donors were infused in mice transplanted with HLA-A2 transgenic murine islets either in the liver, the spleen, under the left kidney capsule (infused 21 days after transplantation, imaged 6 days after infusion) (A), or under the right kidney capsule (infused 49 days after transplantation, imaged 9 days after infusion) (B). The dotted lines in (A) connect the base of the left ear to the base of the tail of the mice as a reference to show the anatomical separation between the liver (to the left of the line) and the spleen and left kidney (to the right of the line). When the right kidney capsule was used as the HLA-A2+ islet transplant site (B), luminescence from A2-CAR T cells could be detected on the right side, but not on the left side of the mouse.


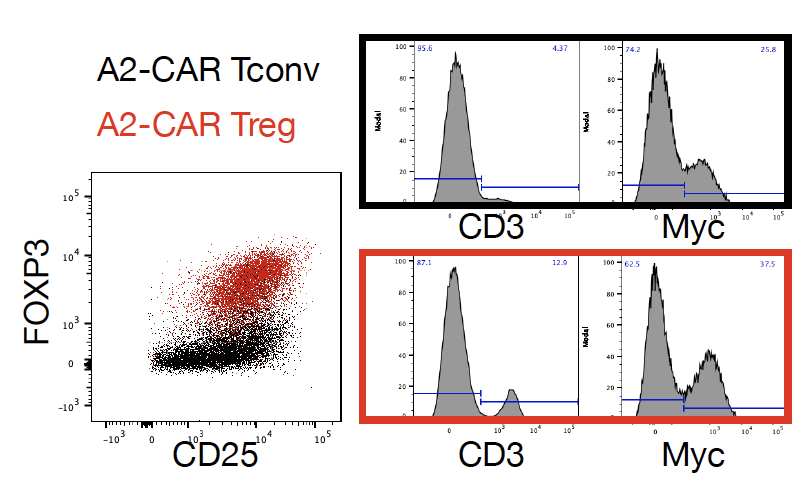


**Supplementary Figure 5: Engineering human A2-CAR^+^TCR^deficient^ Tregs.** CD4^+^CD25^high^CD127^low^ Tregs and CD4^+^CD25^low^CD127^high^ conventional T cells (Tconv) were sorted using FACS. Thereafter, the T-cell receptor (TCR) was deleted using CRISPR/Cas9 and the cells were activated with anti-CD3/CD28 beads. Two days later, cells were lentivirally transduced with A2-CAR. CD25, FOXP3, CD3, and MYC-tag expression were measured on Day 9.


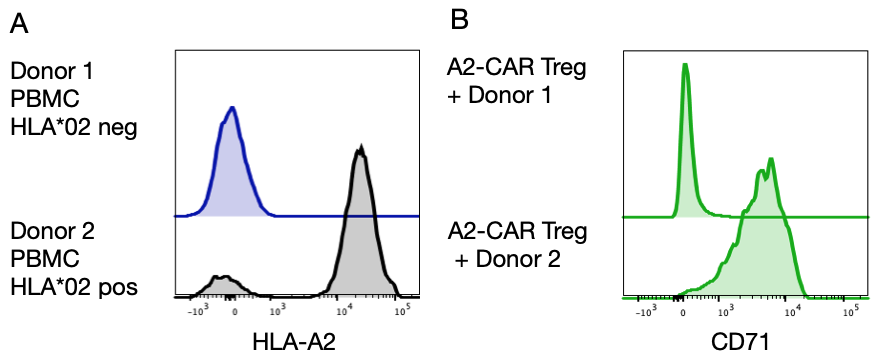


**Supplementary Figure 6: *In vitro* A2-CAR Treg activation by PBMCs from an HLA-A2^+^ donor***.* **(A)** HLA-A2 expression on PBMCs from two donors used for immune reconstitution in NSG mice in Figure 5. **(B)** A2-CAR Tregs were co-cultured for 48 hours at 1:1 ratio with the PBMCs of each donor. CD71 expression was analyzed using flow cytometry.

**Supplementary Table 1: Flow cytometry antibodies used in this study.**

| **Antigen** | **Fluorophore** | **Reactivity** | **Clone** | **Company** | **Location** |
| --- | --- | --- | --- | --- | --- |
| MYC-tag | A647 | Human | 9B11 | Cell Signaling Technologies | Danvers, MA |
| CD69 | PE/Cy7 | Human | FN50 | Biolegend | San Diego, CA |
| CD71 | FITC | Human | CY1G4 | Biolegend | San Diego, CA |
| CD4 | PE/Cy7 | Human | SK3 | Biolegend | San Diego, CA |
| ICOS | BV711 | Human | DX29 | BD Biosciences | San Jose, CA |
| FOXP3 | e450 | Human | PCH101 | eBioscience | San Diego, CA |
| CTLA4 | PerCP-e710 | Human | 14D3 | eBioscience | San Diego, CA |
| EGFR | PE | Human | AY13 | Biolegend | San Diego, CA |
| CD4 | PE | Human | SK3 | Biolegend | San Diego, CA |
| CD8 | PerCP | Human | SK1 | Biolegend | San Diego, CA |
| CD45 | PE/Cy7 | Mouse | 30-F11 | Biolegend | San Diego, CA |
| HLA-A2 | APC | Human | BB7.2 | Biolegend | San Diego, CA |
| HELIOS | FITC | Human | 22F6 | Biolegend | San Diego, CA |
| CD45 | e450 | Human | HI30 | eBioscience | San Diego, CA |
| CD4 | FITC | Human | SK3 | Biolegend | San Diego, CA |
| CD25 | APC | Human | 4E3 | eBioscience | San Diego, CA |
| CD127 | PE | Human | hIL-7R-M21 | BD Biosciences | San Jose, CA |
| Ghost | BV510 |  | Viability dye | Tonbo Biosciences | San Diego, CA |

**Supplementary Table 2: Polymorphic eplets in HLA-A2 and their occurrence in other HLA class I alleles**^1^**.**

| **Eplet** | **Residues** | **Shared with** |
| --- | --- | --- |
| 44RME | 44R45M46E | A3, A11, A23, A24, A25, A26, A29, A30, A31, A32, A33, A34, A43, A66, A68, A69, A74, A80 |
| 62GE | 62G63E | B57, B58 |
| 62GK | 62G66K (74H77D) | none |
| 65RA | 65R69 | A1, A3, A11, A25, A26, A29, A30, A31, A32, A33, A34, A36, A43, A66, A68, A69, A74, A80, B15, B57, B58 |
| 65RK | 65R66K | A34 |
| 66KA | 66K69A | A23, A24, A34 |
| 76VDT | 76V77D80T | A3, A11, A30, A31, A33, A34, A66, A68, A69, A74 |
| 79GT | 79G80T | A1, A3, A11, A26, A29, A30, A31, A33, A34, A36, A43, A66, A68, A69, A74, A80 |
| 80TL | 80T81L | A1, A3, A11, A26, A29, A30, A31, A33, A34, A36, A43, A66, A68, A69, A74, A80, B27, B37, B47 |
| 105S | 105S | A3, A23, A24, A29, A30, A31, A33, A34, A68, A69, A80 |
| 107W | 107W | A69 |
| 109F | 109F | A1, A3, A11, A23, A24, A25, A26, A29, A30, A31, A33, A34, A36, A43, A66, A68, A69, A80 |
| 127K | 127K | A23, A24, A68, A69 |
| 144K | 144K | A1, A3, A11, A24, A36, A68, A69, A80 |
| 144TKH | 142T144K145H | A68, A69 |
| 145KHA | 144K145H149A | A68, A69 |
| 149AH | 149A151H | A1, A3, A11, A24, A36, A68, A69 |
| 150AAH | 149A150A151H | A3, A11, A24, A68, A69 |
| 150AH | 150A151H | A3, A11, A24, A25, A26, A34, A43, A66, A68, A69 |
| 151AHV | 150A151H152V | A24, A68, A69 |
| 184A | 184A | A25, A26, A29, A32, A34, A43, A66, A68, A69, A74 |
| 193AV | 193A194V | A25, A26, A29, A31, A32, A33, A34, A43, A66, A68, A69, A74 |
| 253Q | 253Q | A25, A26, A29, A31, A32, A33, A34, A43, A66, A68, A69, A74, B73, C7, C17 |

^1^ Data contained in this table is extracted from an online HLA Epitope Registry (<https://www.epregistry.com.br/>). In total, 41 eplets are found in HLA-A2. Nine eplets (9F, 66KH, 71HS, 95V, 97R, 99Y, 114H, 116Y, and 207S) are estimated to have low to very low immunogenicity and are excluded from this table. Three eplets (152V, 156L, and 163T) have broad presence in more than 30 HLA-A, -B, and -C alleles and are excluded from this table. Six eplets (44RM, 66K, 77D, 80T, 151H, and 194V) overlap with eplets included in this table and are thus not included for brevity.
